# Towards Sensor-Based Mobility Assessment for Older Adults: A Multimodal Framework Integrating PoseNet Gait Dynamics and InBody Composition

**DOI:** 10.3390/s25185878

**Published:** 2025-09-19

**Authors:** Sinan Chen, Lingqi Kong, Zhaozhen Tong, Yuko Yamaguchi, Masahide Nakamura

**Affiliations:** 1 Center of Mathematical and Data Sciences, Kobe University, 1-1 Rokkodai-cho, Nada, Kobe 657-8501, Japan; masa-n@cmds.kobe-u.ac.jp; 2 Graduate School of Engineering, Faculty of Engineering, Kobe University, 1-1 Rokkodai-cho, Nada, Kobe 657-8501, Japan; 3 Graduate School of Economics, Faculty of Economics, Kobe University, 2-1 Rokkodai-cho, Nada, Kobe 657-8501, Japan; 219e115e@gsuite.kobe-u.ac.jp; 4 Graduate School of Health Sciences, Kobe University, 7-10-2 Tomogaoka, Suma, Kobe 654-0142, Japan; y.yuko@port.kobe-u.ac.jp; 5 RIKEN Center for Advanced Intelligence Project, 1-4-1 Nihonbashi, Chuo-ku, Tokyo 103-0027, Japan

**Keywords:** gait analysis, computer vision, health monitoring, composition metrics, mobility and health correlation

## Abstract

The acceleration of global population aging has driven a surge in demand for health monitoring among older adults. However, traditional mobility assessment methods mostly rely on invasive measurements or laboratory-grade equipment, making it difficult to achieve continuous monitoring in daily scenarios. This study investigated the correlation between dynamic gait characteristics and static body metrics to enhance the understanding of elderly mobility and overall health. A sensor-based framework was implemented, which utilizes the *Short Physical Performance Battery (SPPB)*, combined with *PoseNet* (a vision-based sensor) for dynamic gait analysis, and the *InBody* bioelectrical impedance device for static body composition assessment. Key variables comprised the dynamic metric *mean directional shift* and static metrics, including *skeletal muscle index (SMI)*, *skeletal muscle mass (SMM)*, *body fat percentage (PBF)*, *visceral fat area (VFA)*, and *intracellular water*. Nineteen elderly participants aged 60–89 years underwent assessments; among them, 16 were males (84.21%), and 3 were females (15.79%), 50% were in the 80–89 age group, 95% did not live alone, and 90% were married. Dynamic gait data were analyzed for center displacement and horizontal directional shifts. A Pearson correlation analysis revealed that the mean directional shift positively correlated with *SMI* (ρ=0.561, p<0.01), *SMM* (ρ=0.496, p<0.01), and *intracellular water* (ρ=0.497, p<0.01), highlighting the role of muscle strength in movement adaptability. Conversely, negative correlations were found with *PBF* (ρ=−0.256) and *VFA* (ρ=−0.342, p<0.05), suggesting that greater fat mass impedes dynamic mobility. This multimodal integration of dynamic movement patterns and static physiological metrics may enhance health monitoring comprehensiveness, particularly for early sarcopenia risk detection. The findings demonstrate the framework’s potential, indicating mean directional shift as a valuable dynamic health indicator.

## 1. Introduction

The global acceleration of population aging has led to a substantial increase in the demand for eldercare services and health monitoring systems, placing considerable pressure on both public medical infrastructure and family caregiving systems [1,2]. Against this backdrop, ensuring efficient assessment and the continuous monitoring of elderly individuals’ health status has emerged as a critical societal challenge. In response, researchers and policymakers have increasingly turned to intelligent healthcare solutions [3,4].

The rapid development and integration of the *Internet of Things (IoT)* [5,6,7] and *Information and Communication Technology (ICT)* into eldercare systems has enabled the convergence of digital technologies and personalized care strategies [8]. In this context, wearable sensor technology, as an important part of intelligent health monitoring, is widely used in the activity tracking and physiological indicator collection of older adults, playing a positive role in improving remote monitoring capabilities [9,10]. However, the development of such integrative approaches has been constrained in part through the reliance on contact-based measurement techniques, which may be invasive, impractical for continuous use, or difficult to implement in vulnerable populations, particularly among older adults. In contrast, noncontact technologies, especially vision-based sensing solutions, are gradually becoming more suitable alternative pathways for vulnerable populations due to their low invasiveness and excellent daily adaptability [11,12]. In particular, vision-based gait assessment is considered a promising method for assessing physical function in older adults due to its ability to perform continuous monitoring under noninvasive conditions and to avoid dependence on contact sensors or clinical settings [13,14,15]. Despite recent technological advancements, the relationship between observable gait characteristics and underlying musculoskeletal health remains insufficiently examined [16]. Most existing studies address gait performance and physiological indicators as distinct research domains, resulting in a limited understanding of their interrelated mechanisms. Although several studies have attempted to bridge this gap, for example, by incorporating musculoskeletal modeling with joint kinematics, or by employing three-dimensional joint displacement metrics to classify neuromuscular disorders, such efforts are typically confined to disease-specific diagnostic contexts or narrowly defined participant populations [17,18,19].

To address these limitations, the present study proposed an integrated sensor instrumentation framework that leverages noninvasive pose-estimation-sensing technology. By combining this with gait feature data and physiological body composition data, the framework aimed to develop a novel method for assessing musculoskeletal health in older adults. To evaluate the feasibility and validity of this integration, a pilot study was conducted involving nineteen elderly participants. All feature extraction and statistical processing were fully automated, facilitating potential scalability to broader remote health monitoring or community-based screening applications. In future work, we aim to expand this approach to larger populations and assess its robustness across diverse real-world settings. The main contribution of this study lay in constructing a dynamic motion metric, namely center displacement (defined as the movement distance of the human center of mass (CoM) in the pixel space), using data extracted via the non-invasive computer vision model PoseNet, and subsequently verifying the correlation between this dynamic motion metric and the physiological indicators related to the physical function of the elderly through correlation analysis. This approach may reduce dependence on laboratory-grade instrumentation while enabling the development of personalized, real-time feedback systems to support healthcare decision-making and early interventions. Ultimately, our goal was to contribute to the development of intelligent eldercare environments where mobility and health status can be continuously monitored using noninvasive technologies.

The aim of this study was to analyze correlations between dynamic movement metrics and static physiological metrics (e.g., *SMI*, *SMM*, and *PBF*) by integrating non-invasive gait dynamic feature detection based on PoseNet and static body composition data measured using InBody devices and, further, to develop a non-invasive, multimodal method applicable to musculoskeletal health assessment and early sarcopenia risk detection for older adults, with the goals of enhancing the comprehensiveness of health monitoring for older adults, enabling non-invasive monitoring in daily scenarios, and reducing reliance on traditional invasive measurements or laboratory-grade equipment.

## 2. Related Work

Human gait is a complex, multidimensional process resulting from the dynamic interaction of biomechanical structures, neural control mechanisms, environmental contexts, and individual-specific factors [20,21]. Among its quantifiable characteristics, gait variability has been increasingly recognized as a sensitive indicator of musculoskeletal health, fall risk, and age-related functional decline, including neurodegenerative conditions such as Parkinson’s disease [22,23]. This variability is commonly evaluated through parameters such as directionality, symmetry, and stability [24,25,26,27].

### 2.1. Vision-Based Gait Analysis: From Pose Estimation to Health Assessment Utility

Existing studies have demonstrated that gait patterns contain structured and individual-specific characteristics that can be captured using visual representations such as point-light displays and reflective markers attached to human joints. These findings have shown that gait carries discriminative features that are consistent across individuals and can be extracted under simplified visual conditions. Although these methods were initially developed for biometric identification, they provide important empirical evidence supporting the use of vision-based techniques to extract meaningful gait features. This foundation has informed subsequent research aiming to utilize visually observable gait characteristics as indicators of physical condition and musculoskeletal health [28,29]. With the rapid advancement of visual sensing technologies, including RGB cameras, depth sensors, and infrared imaging devices, gait analysis has entered a new stage of development [30]. These technologies offer noninvasive and high-resolution means of capturing human movement and have increasingly been used to complement conventional sensor-based systems [31,32,33,34,35]. Recent studies have demonstrated that vision-assisted frameworks, such as those incorporating binocular cameras for sensor-to-segment alignment, can significantly improve the accuracy of joint angle estimation in inertial sensor-based gait analysis [36]. For tremor, a complex and common neurological symptom, applying Mediapipe to clinical videos of patients with essential tremor has confirmed its high clinical validity, consistency with gold-standard measurements, and ability to capture features missed when traditional scores are used [37]. In parallel, the integration of computer vision and deep learning techniques has enabled pose estimation algorithms to extract skeletal keypoints from standard video inputs, offering a promising, contactless approach to mobility assessment in naturalistic settings [38,39,40].

In addition to three widely used real-time human pose estimation (HPE) models for gait analysis, namely PoseNet, OpenPose, and MoveNet, frameworks including Mediapipe and DeepLabCut present distinct strengths and limitations for geriatric health monitoring. Mediapipe supports 33 skeletal keypoints (including fine- grained features like fingers and toes), achieves a greater real-time frame rate (60 fps) than PoseNet, and performs better in occluded scenarios (e.g., heavy clothing on elderly individuals), with an error rate below 8%—outperforming PoseNet’s 10–15% accuracy decay [37]. However, its dependence on Google’s Software Development Kit (SDK) and redundant multi-modal data (e.g., facial or hand tracking) increases deployment complexity in community settings, reducing accessibility for non-specialized care providers. DeepLabCut offers customization for keypoint selection and reaches 98.5% accuracy after scenario-specific training, but its offline analysis workflow (real-time frame rate < 5 fps) and need for extensive annotated gait data (2–4 weeks of sample labeling) make it unsuitable for the continuous dynamic monitoring of elderly mobility. OpenPose can detect 25 keypoints (including detailed foot features) and has been validated in neurological gait disorder diagnosis (e.g., Parkinson’s disease) [41], yet it requires high-performance GPU support (VRAM > 4 GB) and strict environmental control (e.g., solid-color backgrounds), limiting its scalability beyond laboratory or clinical settings. Among these frameworks, *PoseNet* was selected for this study due to its lightweight architecture, compatibility with mobile deployment, and minimal computational requirements—attributes that align with the need for accessible, continuous geriatric health monitoring in non-clinical community settings.

Among the *human pose estimation (HPE)* models in this domain, *PoseNet*, OpenPose, and MoveNet have demonstrated strong performance in real-time applications. *PoseNet*, in particular, is notable for its lightweight architecture and mobile deployment compatibility, making it especially suitable for elderly health monitoring outside of clinical environments. Comparative studies on mobile platforms have shown that *PoseNet* achieves the greatest pose estimation accuracy (97.6%), with minimal computational variance and strong real-time performance, underscoring its suitability for applications requiring both precision and stability [42,43]. When integrated with pose estimation algorithms, it offers a scalable solution for remote health monitoring. PoseNet’s ability to autonomously capture and process gait sequences with minimal user intervention represents a major advancement in practical geriatric assessment [44,45].

### 2.2. Quantifying Functional Decline: Gait Features as Health Indicators

Visual gait features are increasingly recognized not only as indicators of mobility but also as surrogate markers for broader aspects of physiological and neuromuscular health. Empirical studies have demonstrated the feasibility of applying vision-based systems to functional assessments in older adults. Low-cost configurations, such as Raspberry Pi devices with multi-camera setups, have successfully measured gait speed, balance, and mobility in both healthy and clinical populations, including individuals undergoing cancer treatment. These systems have achieved over 97% temporal accuracy in commonly used functional tests such as the *SPPB* and Timed Up and Go (TUG), demonstrating strong potential for reliable and scalable gait assessment [46]. As a widely used clinical tool for evaluating lower extremity function, the *SPPB* specifically targets core dimensions of physical performance, including balance, gait speed, and chair stand ability, and it has shown predictive validity for adverse outcomes such as mortality and institutionalization [47,48,49].

### 2.3. Integrating Static Physiology with Dynamic Mobility Metrics

The decline in musculoskeletal health is a common consequence of aging and a key contributor to reduced mobility, increased fall risk, and diminished quality of life in older adults [50,51,52,53]. Impairments in lower extremity strength and balance significantly limit independence, while they are also associated with greater rates of hospitalization [54,55]. As a response, the assessment of skeletal muscle mass and quality has become a central focus of geriatric health monitoring [56]. Sarcopenia, characterized by the age-related loss of muscle mass and strength, is a well-established risk factor for frailty, impaired mobility, and loss of independence. Sarcopenia in older adults refers to the loss of skeletal muscle mass and function, caused by various factors (such as cellular, neural, metabolic, and hormonal factors) [57,58]. By the age of 80, the muscle mass of older adults has been shown to decrease by 40% compared to when they were 20 years old [59].

Standard methods such as *bioelectrical impedance analysis (BIA)* and *dual-energy X-ray absorptiometry (DEXA)* are widely used to quantify body composition, including *SMM*, *SMI*, *PBF*, and *fat-free mass (FFM)* [60]. In clinical and research settings, *InBody* devices are particularly valued in both clinical and research settings due to their portability and operational simplicity. They are commonly used in health screenings and aging-related studies to identify individuals at risk of physical decline or metabolic imbalance [61]. However, measures of muscle mass alone do not reflect neuromuscular coordination, adaptive control, or dynamic postural stability, all of which are essential for safe ambulation.

Despite parallel advancements in visual gait analysis and physiological assessment, few studies have attempted to integrate these domains. This limitation has led to increased interest in integrating static physiological assessments with dynamic mobility metrics to better understand aging-related functional deterioration. Early findings indicate associations between impaired gait and muscle weakness; particularly in the lower limbs, muscle deficits can lead to an altered center of mass displacement and irregular gait patterns [62]. Changes in gait trajectory, asymmetrical weight transfer, and deviations in stride length have been interpreted as indirect indicators of musculoskeletal impairment [63]. Furthermore, visual gait data may be particularly useful in detecting subtle declines not readily observable through static measurements. This potential for early detection positions vision-based analysis as a valuable complement to traditional muscle health assessments.

## 3. Experimental Methodology

The remainder of this paper is structured as follows: Section 3 outlines the methodology, including data collection, feature extraction, and correlation analysis. Section 4 presents the results and interpretation. The final section discusses the findings, limitations, and future research directions.

### 3.1. Purpose and Environmental Setup

To support the study on the correlation between dynamic gait characteristics and static body composition metrics, this research defined the necessary experimental equipment configuration, environmental control, data collection specifications, and quality calibration standards: A Sanwa Supply webcam was used for gait data collection, equipped with a 150° ultra-wide-angle lens and a maximum frame rate of 30 fps to ensure data integrity. The experiment was conducted in a controlled laboratory environment with stable lighting and a plain background, where participants were required to wear light-colored, non-loose clothing and avoid accessories. Recordings were taken primarily from a frontal view, supplemented with a slight sagittal view. The detailed parameters of the aforementioned experimental setup can be found in Table 1. To reduce the experimental burden on participants, the study adhered to a voluntary participation principle, allowing participants to withdraw at any stage. Furthermore, all participants provided written informed consent before the tests. The experimental data were stored in an encrypted database, used solely for analysis in this study, and not disclosed to any third party, except the experiment administrators. The study design and implementation were approved by the Kobe University Ethics Committee (Approval Number: 1249). To enhance the data comparability within the study cohort, this study adopted the fixed experimental settings presented in Table 1, aiming to minimize the interference of other irrelevant variables (noise) during the experiment and ensure the accuracy and reliability of subsequent data analysis.

The experiment was conducted on 25 July 2024, from 10:00 a.m. to 2:00 p.m., in a controlled laboratory environment. To ensure the stability and accuracy of data collection, the laboratory’s lighting conditions were strictly controlled, and the positions of the recording devices were precisely calibrated. Fast-frame-rate cameras were utilized to capture participants’ dynamic gait trajectories during the *SPPB* test, providing high-resolution raw data for gait feature extraction. Simultaneously, static body metrics, including *SMM*, body fat percentage, and *BMI*, were measured using the *InBody* device. All participants completed the experiments in individual testing sessions to ensure the independence and accuracy of data collection. Each participant underwent two rounds of *SPPB* tests and *InBody* measurements, with dynamic gait testing and static metric measurement conducted separately to minimize potential interference. The demographic and lifestyle characteristics of these participants are summarized in Table 2. A total of 19 participants were recruited for this experiment, including 16 males (80%) and 4 females (20%). The participants ranged in age from 60 to 89 years, with 50% aged between 80 and 89, 20% aged between 70 and 79, and 30% aged between 60 and 69. Regarding living arrangements, the majority of participants (95%) lived with others, while only one participant lived alone (5%). In terms of marital status, 90% of the participants were married, while 10% were unmarried. Regarding employment status, 30% of participants were employed, and 70% were unemployed.

### 3.2. Overview of Proposal Method

To reduce the experimental burden on participants, the study adhered to a voluntary participation principle, allowing participants to withdraw at any stage. Furthermore, all participants provided written informed consent before the tests. The experimental data were stored in an encrypted database, used solely for analysis in this study, and not disclosed to any third party except the experiment administrators. The study design and implementation were approved by the Kobe University Ethics Committee (Approval Number: 1249).

**Figure 1:**
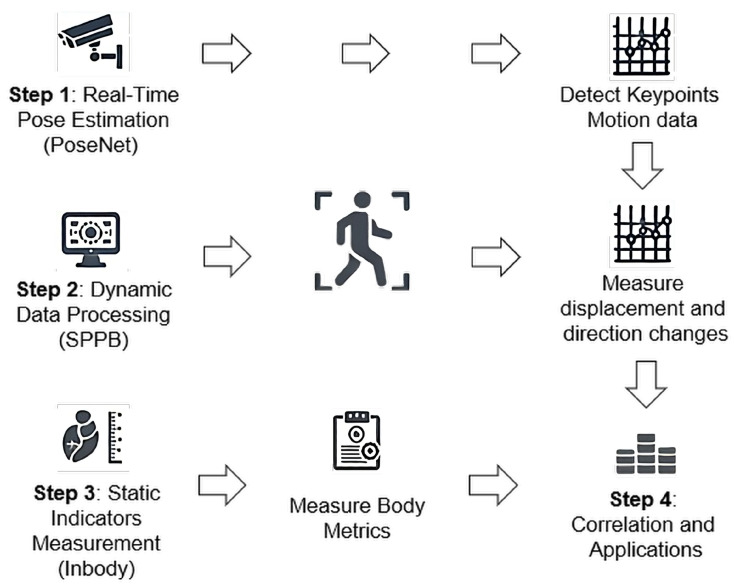
Flowchart of Integrating SPPB, InBody Measurements, and Motion Analysis: Step-by-Step Process.

**Figure 2:**
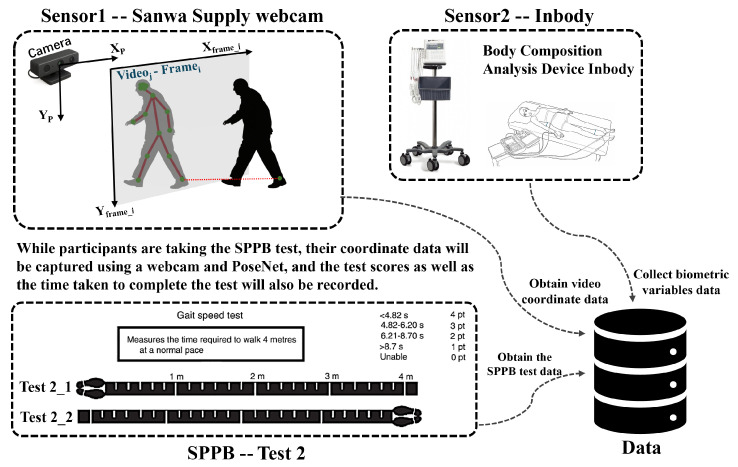
Schematic diagram of experimental environment setup.

To explore the potential relationship between dynamic body characteristics and static physical metrics, an integrated experimental and data analysis approach was employed by this study, as outlined in Figure 1. The experiment utilized gait data collected through the *SPPB* gait test, combined with body composition metrics obtained from *InBody* measurements, as shown in Figure 2, to reveal the connections between dynamic and static data. The experimental procedure was divided into five main steps:

### 3.3. STEP 1: PoseNet Model Detection

**Figure 3:**
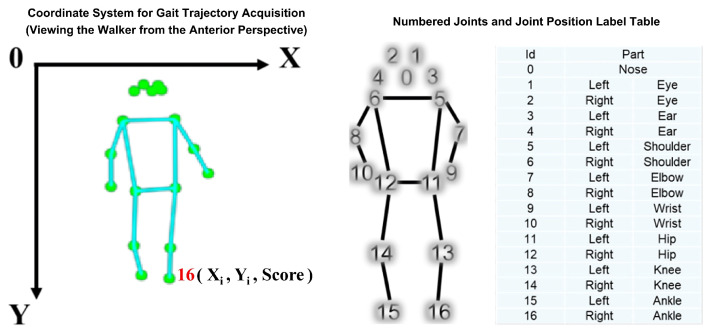
Coordinate acquisition and joint labeling for human pose recognition based on PoseNet.

First, the lightweight and efficient *PoseNet* model was employed to capture real-time dynamic gait trajectories of participants using camera equipment. For each frame, the model detected and extracted the two-dimensional coordinates and confidence scores of 17 key body points. This process transformed dynamic gait characteristics into structured data, providing precise raw information essential for the subsequent calculation of dynamic characteristics, feature extraction, and correlation analysis with static metrics. In Figure 3, the left side presents the coordinate system (CS) and the stick figure, which intuitively demonstrates the method of acquiring coordinates when observing a pedestrian; the right side consists of two parts: one part depicts the numbered joints, and the other part (a table) labels the meaning corresponding to each recognized joint position.

**Figure 4:**
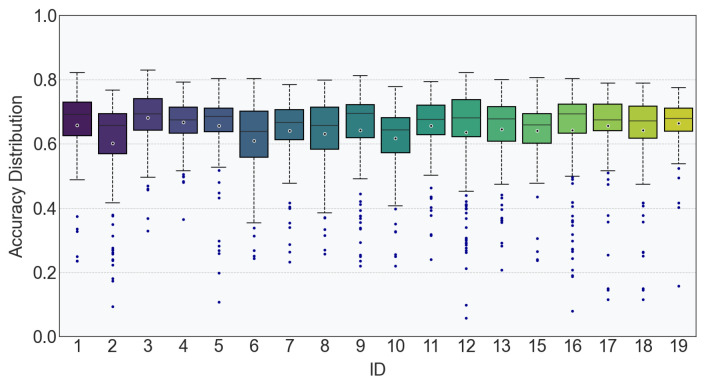
Average accuracy of Posenet-extracted centroid-related coordinate date.

To verify the reliability of *PoseNet* for gait analyses of the elderly under the specific experimental settings of this study, we further validated the accuracy of the confidence values for the centroid-related coordinate data (i.e., the coordinates of five body parts: nose, right shoulder, left shoulder, left hip, and right hip) extracted via *PoseNet*. As shown in Figure 4, the average values ranged from 0.59 to 0.68, predominantly clustering within the 0.60–0.67 interval; the median values spanned from 0.63 to 0.70, with most participants’ medians falling between 0.66 and 0.69; the maximum values concentrated in the range of 0.77–0.83, for which participant ID3 achieved maximum value (0.83), and participants ID2 and ID14 achieved the lowest value (0.77); the first quartile (Q1) was between 0.54 and 0.64, and the third quartile (Q3) was between 0.68 and 0.74. The relatively concentrated distribution of these data indicates that the accuracy of *PoseNet* in extracting centroid-related coordinate data for gait analyses of the elderly in this study exhibits certain stability, suggesting that *PoseNet* achieves a certain level of reference value in such application scenarios.

### 3.4. STEP 2: Outlier Handling of Center Displacement

#### 3.4.1. Center Displacement: Overall Trends and Anomaly Analysis

As the core dynamic indicator in this study for reflecting the gait stability and dynamic adjustment ability of older adults, center displacement was defined as the movement distance of the body’s center of mass (CoM) in the pixel space between consecutive frames. The specific calculation procedure is detailed in Section 3.5.2. The core value of this indicator resides in two aspects: On the one hand, its pixel-level quantitative results can directly capture the subtle fluctuations in the body’s center of gravity during gait, thereby avoiding the interference of traditional contact-based sensors on movement. On the other hand, the gait stability of older adults can be comprehensively assessed by quantitatively monitoring the displacement characteristics of center displacement. A smaller displacement value indicates a stable gait, whereas a larger displacement value may imply body imbalance or suboptimal physical health status. This holds significant reference value for evaluating the muscle function of older adults, and it provides a key basis for assessing their physical health conditions.

**Figure 5:**
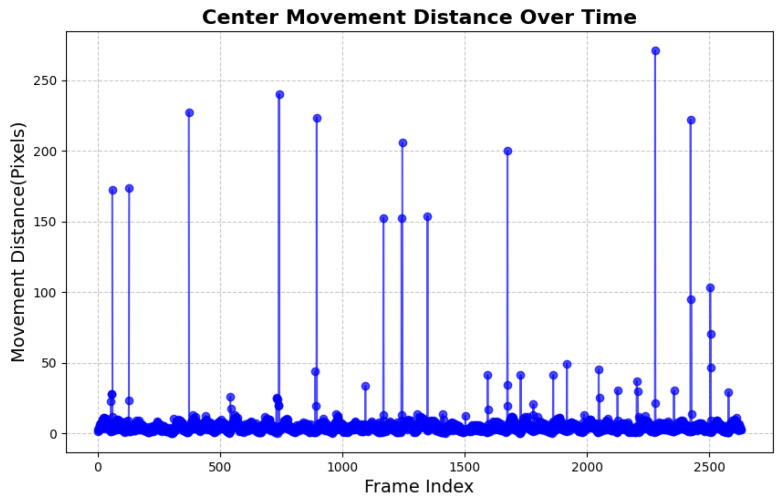
Time-series representation of center movement distance. The vertical axis represents the movement distance in pixels, while the horizontal axis represents the frame index of the recorded motion data. Peaks in the graph indicate significant shifts in the center position.

Figure 5 illustrates the temporal variation in center displacement, highlighting the dynamic characteristics of the gait data. In the figure, the horizontal axis (*Frame Index*) represents the sequence of recorded time frames, ranging from 0 to 2500, which corresponds to the complete frame range captured during the experiment. The vertical axis (*Movement Distance in Pixels*) indicates the displacement of the center point in each frame. From the overall distribution, most frames exhibit center displacements concentrated within a low range (approximately 0–20 pixels), suggesting that participants maintained a relatively stable motion state for the majority of the experiment. However, distinct peaks were observed in some frames (displacement greater than 50 pixels), which typically reflect abrupt movements or other anomalous events during the experimental process. By examining the distribution, we preliminarily confirmed that the majority of the data points lie within a stable range, with a certain proportion of extreme values (outliers). To improve the accuracy and consistency of data analysis, these anomalies require further processing. Subsequent analyses will filter these outliers to ensure that the research results are more reliable and interpretable.

#### 3.4.2. Center Displacement: Trend and Outlier Analysis

**Figure 6:**
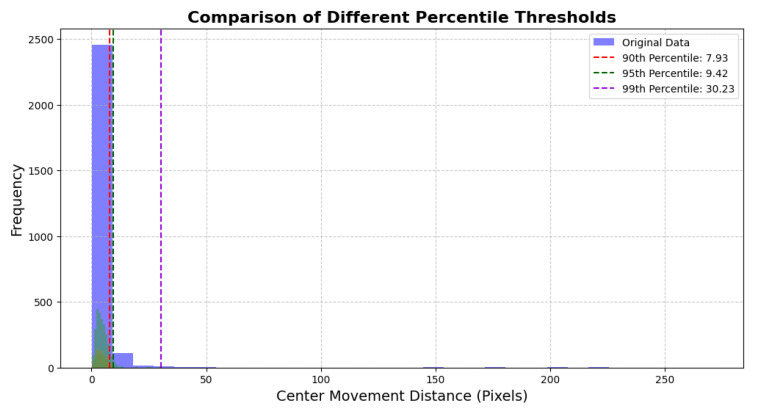
Comparison of different percentile thresholds for center movement distances. The X-axis represents the movement distance (in pixels), and the Y-axis represents the frequency of occurrences. The Dashed lines indicate the thresholds at the 90th, 95th, and 99th percentiles.

This subsection describes how, to ensure the accuracy and robustness of data analysis, we processed outliers in the core variable (center displacement) data using the percentile threshold method. Specifically, the 90th, 95th, and 99th percentiles of the data were calculated, and values exceeding these thresholds were identified as potential outliers, as illustrated in Figure 6. The choice of the percentile method was based on the following considerations.

**Adaptation to Tail Distribution Characteristics:**

The percentile method is particularly suited for datasets with long-tail distributions. Unlike the *IQR* method, it emphasizes the proportion of tail outliers. In Figure 6, the data distribution exhibits a right-skewed tail, making the percentile method more effective in capturing tail-end outliers.

**Robustness to Asymmetric Distributions:**

Compared to the *IQR* method, which relies on symmetry assumptions, the percentile method is not constrained by distribution symmetry. Since the distribution in Figure 6 is not perfectly symmetric, the percentile method offers greater flexibility in defining the outlier thresholds.

**Flexibility in Graded Processing:**

The percentile method allows for the selection of different percentiles (90%, 95%, or 99%) based on research needs, enabling adjustable outlier filtering. The specific percentile thresholds are shown in Figure 6, with the 90th, 95th, and 99th percentiles calculated as 7.93, 9.42, and 30.23, respectively, offering multiple possibilities for outlier processing.

Figure 6 presents the original distribution of center displacement data and the effects of outlier cleaning using different percentile thresholds. The blue histogram represents the original data distribution, while the green, orange, and purple dashed lines represent the cleaned data distributions based on the 90th, 95th, and 99th percentiles, respectively. The distribution of center displacement exhibits a significant long-tail phenomenon, with the top 10% to 1% of data points deviating far from the main distribution. These values are likely influenced by equipment errors or incidental movements, potentially interfering with the analysis of the relationship between movement variation and body metrics.

**Selection of the 95th Percentile:**

For this study, the 95th percentile (green dashed line, threshold = 9.42) was selected as the outlier removal standard to eliminate the top 5% of data points, and this choice was fully justified by the alignment of data characteristics with research needs: First, as shown in Figure 6, the center displacement data presents a significant right-skewed long-tail distribution, with the top 5% of data points clearly deviating from the main distribution; cross-referencing with raw video recordings of participants’ gait tests confirms that these points correspond to non-standard movement behaviors (e.g., sudden body sway from accidental slips, temporary posture adjustments for camera adaptation) or equipment noise (e.g., pixel jumps from frame freezing), all unrelated to the stable gait characteristics targeted in this study, proving that the 95th percentile accurately identifies non-informative outliers. Compared to the 90th percentile (threshold = 7.93), which may eliminate the top 10% of data and lose valid high-displacement gait data, reflecting real movement variability (e.g., slight balance adjustments during normal walking), the 95th percentile avoids over-filtering and preserves meaningful movement variation features; compared to the 99th percentile (threshold = 30.23), which only removes the top 1% of data and fails to address most equipment-induced noise (e.g., short-term continuous frame distortion), the 95th percentile eliminates more such noise, evidenced by the more concentrated cleaned data distribution in Figure 6, reducing interference with subsequent correlation analyses. To verify this outlier treatment’s reliability, the core logic of sensitivity analysis was considered: evaluating the impact of different percentile thresholds on key correlation patterns confirmed that the 95th percentile did not distort the critical associations between mean directional shift and core body metrics and effectively balances data cleanliness and information retention. Regarding its impact on study results, analyses using 95th-percentile-processed data were more balanced, avoiding weakened true correlations from over-filtering and reducing residual noise’s interference with result reliability. The cleaned data distribution was more centralized while retaining sufficient movement variation, providing a solid foundation for analyzing correlations between movement changes and body metrics.

### 3.5. STEP 3: Dynamic Data Collection and Feature Extraction

Second, *SPPB* was employed to capture dynamic gait data, with a specific focus on its walking test component. The *SPPB* protocol comprehensively assesses lower limb function through three domains: balance tests, walking tests, and chair-rise tests. During the walking test phase, participants’ real-time gait trajectories were recorded using camera equipment integrated with the *PoseNet* model. The detailed experimental design is as follows.

#### 3.5.1. Walking Test of SPPB

In the walking test, participants were instructed to walk a straight, 4-m path at a normal pace. The time taken to complete the walk was measured twice, and the average of the two measurements was recorded. Based on predefined time intervals, the walking speed (measured in seconds) was scored on a scale of 0 to 4, with greater scores indicating better physical performance.

**Figure 7:**
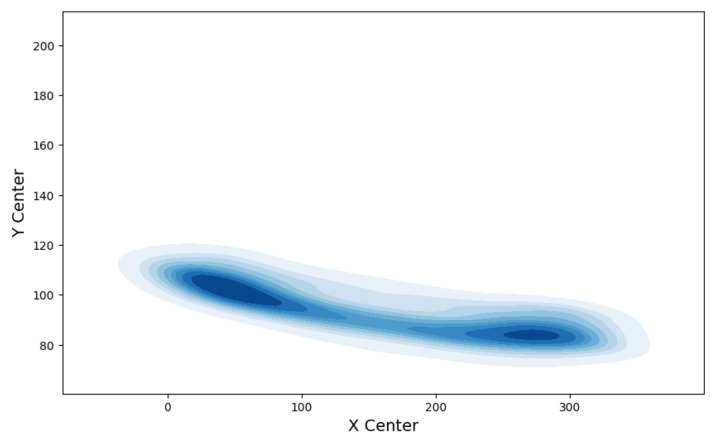
Walking path trajectory density plot for all participants (X-axis = X-range of center-of-mass coordinates for all participants during movement [horizontal direction]; Y-axis = Y-range of center-of-mass coordinates for all participants during movement [vertical direction]; color intensity = distribution density).

To provide a more intuitive visualization of participants’ dynamic performance during walking, a path density plot was used to analyze the recorded walking trajectories with center-of-mass coordinates as core data. Figure 7 illustrates the distribution of the central trajectories recorded during the walking tests for all participants, where the X-axis and Y-axis in the figure represent the X and Y coordinates of the participants’ center of mass during movement, respectively. The density in the plot represents the concentration of trajectory points on this two-dimensional center of mass coordinate plane, where darker blue areas indicate greater trajectory point density. The dense areas (dark blue regions) show that the distribution of most participants’ center of mass positions converged in these high-density regions, indicating that they maintained relatively stable and consistent center-of-mass movement paths during walking, which in turn reflects the stability of the overall walking path. This center-of-mass trajectory distribution feature can supplement and verify the stability of participants during movement from the perspective of “dynamic changes in spatial position”—compared with indicators such as walking speed that only reflect “speed,” the distribution density of center-of-mass coordinates can more directly reflect the control ability of the body’s core position during walking, complementing the “gait stability” assessment dimension in standard gait characteristics. However, some low-density areas (light blue regions with sparse trajectory points) also appeared in the figure. The formation of these areas may stem from two reasons: first, changes in the distance between participants and the camera during walking, which resulted in partial deviations in center-of-mass trajectories; and second, differences in gait behavior among individuals (such as variations in step width and center-of-mass transfer amplitude), leading to a more scattered distribution of center-of-mass trajectories.

#### 3.5.2. Key Point Extraction and Center-of-Gravity Calculation

**Step 3.1: Selection of Key Points from PoseNet Output**

Key points, including the nose (0), left shoulder (5), right shoulder (6), left hip (11), and right hip (12), were selected from the *PoseNet* model output. Using the coordinates of these key points, the *center of mass (CoM)* coordinates $x_{center}$, $y_{center}$ for each frame were calculated. The calculation formula was as follows:

(1) $$x_{center}=\frac{\sum_{i\in K}x_{i}}{\left|K\right|},y_{center}=\frac{\sum_{i\in K}y_{i}}{\left|K\right|}$$

where $x_{i}$, $y_{i}$ represents the coordinates of the *i*th key point, and |K| is the total number of selected key points.

**Step 3.2: Calculation of Center Displacement**

The center displacement, which reflects the *CoM* movement distance, was calculated using the Euclidean distance between the *CoM* coordinates of consecutive frames. The formulas were given as follows:

(2) $$euclid\_dis_{i,j}=\sqrt{{\left(x_{i}-x_{j}\right)}^{2}+{\left(y_{i}-y_{j}\right)}^{2}}$$

(3) $$center\_dis_{i}=\sqrt{{\left(x_{center,i}-x_{center,i-1}\right)}^{2}+{\left(y_{center,i}-y_{center,i-1}\right)}^{2}}$$

where $\left(x_{center,i},y_{center,i}\right)$, and $\left(x_{center,i-1},y_{center,i-1}\right)$ are the *CoM* coordinates at time *i* and i−1, respectively.

**Step 3.3: Calculation of Directional Shift**

The directional shift in *CoM* movement was computed as follows [64]:

(4) $$\theta_{t}=\arctan\left(\frac{y_{center,i}-y_{center,i-1}}{x_{center,i}-x_{center,i-1}}\right)$$

where $\theta_{t}$ represents the angular shift in the *CoM* movement direction between consecutive frames.

**Step 3.4: Aggregation of Dynamic Characteristics**

To summarize the dynamic characteristics, the statistical properties of horizontal directional shifts, such as mean and variance, were computed. Meanwhile, the metric unit of horizontal directional shifts was standardized to be consistent with that of overall displacement, both denoted as pixel/frame. This unit specifically represents the pixel distance of the CoM’s horizontal directional shift and overall displacement change between consecutive frames (1 frame).

In the experiment, participants completed tests at a fixed camera position with essentially identical shooting distances. This experimental design ensured the accuracy and reliability of the current dataset. This experimental design aimed to reduce the interference of shooting parameter differences on the data and provide a foundational support for the accuracy and reliability of the current dataset. However, it should be noted that this study has not yet verified the “accuracy” and “reliability” through quantitative methods (such as setting clear error thresholds, conducting statistical verification, etc.), which constitutes a limitation of the current experimental design. To address the issue of data comparability under different shooting distances and experimental setups in subsequent studies, corresponding improved methods are proposed. For example, scale normalization is adopted to perform scale normalization processing on data obtained from different camera distances; the Kalman filter (KF) and dynamic time warping (DTW) are used to eliminate noise in motion data and align spatially parallel body symmetry points, etc. These methods collectively achieve unified calibration and correction of multi-scenario data.

### 3.6. STEP 4: Static Metric Measurement

Third, the measurement of static body metrics primarily relied on the *InBody* device, which utilizes *BIA* to accurately assess individual body composition characteristics. The specific parameters measured include *SMM*, *PBF*, *BMI*, and other key indicators reflecting body composition. The principle of *InBody* measurement is based on the impedance properties of different tissues to electrical currents. For instance, muscle, with its high water content, conducts electricity more effectively than fat, which has lower conductivity. By employing multi-frequency current measurements, the device is able to differentiate and quantify the composition of various tissues, providing multidimensional metrics such as *body weight*, *FFM*, and *total body water (TBW)*. The specific measured indicators are described in detail in Table 3.

Table 4 presents a statistical summary of the *InBody* measurements. Key indicators include *SMI* (mean: 8.132; range: 5.5–10.2) and *SMM* (mean: 25.653; range: 15.8–32.3), which are crucial for evaluating skeletal muscle health and are directly linked to sarcopenia and muscle function. *FFM* (mean: 47.416) and *PBF* (mean: 19.7) reflect fat-free mass and body fat percentage, respectively. The former is used to assess the health of body composition, while the latter was associated with obesity risk. *VFA* (mean: 46.316) reveals visceral fat distribution, and *BMI* (mean: 22.695) provides a foundational assessment of body weight status.

### 3.7. STEP 5: Pearson Correlation Analysis

In the final phase of this study, a correlation analysis was conducted to investigate the relationship between dynamic gait characteristic variables and static body metrics, based on integrated data that combined center-of-mass parameters, SPPB test results, and static body metrics using participants’ IDs as the key value. This integrated approach, which merges dynamic movement data with static physiological indicators, aims to explore the potential application of body movement characteristics in health management and disease prevention. The *Pearson correlation coefficient (PCC)* was used as the primary measure, and its formula is expressed as follows:

(5) $$r=\frac{\sum_{i=1}^{n}\left(x_{i}-\bar{x}\right)\left(y_{i}-\bar{y}\right)}{\sqrt{\sum_{i=1}^{n}{\left(x_{i}-\bar{x}\right)}^{2}}\sqrt{\sum_{i=1}^{n}{\left(y_{i}-\bar{y}\right)}^{2}}},$$

To determine the appropriateness of using the Pearson correlation analysis (which requires continuous variables and adherence to a normal distribution as key assumptions), all body composition-related indicators in this study were first verified for their data properties and normality. All indicators involved are continuous variables that meet the basic data type requirement of Pearson correlation. Normality testing was conducted using the Shapiro–Wilk test (significance level set to α=0.05; the conformity to normal distribution is indicated when p>0.05). As shown in Table 5, among all tested indicators, only the “FFM Control” variable did not conform to a normal distribution (p=0.0002), while the remaining 23 indicators (including ICW, ECW, BMI, VFA, etc.) all achieved *p*-values >0.05, confirming their compliance with the normal distribution assumption. In summary, the majority of the core continuous indicators in this study meet the normality requirement of Pearson correlation analysis. Thus, the Pearson correlation coefficient was selected for a subsequent correlation analysis to explore the associations between variables.

The above *r* represents the Pearson correlation coefficient, which ranges from [−1,1]. A value of r>0 indicates a positive correlation, r<0 indicates a negative correlation, and r=0 denotes no correlation. The terms $x_{i}$ and $y_{i}$ refer to the observed values of the dynamic gait characteristic variable and static body metric variable, respectively. $\bar{x}$ and $\bar{y}$ denote their respective means, and *n* represents the sample size.

To further examine the significance of the correlations, the study calculated the *p*-value of the correlation coefficient to assess statistical significance. The hypothesis testing framework was as follows:

- Null hypothesis $\left(H_{0}\right)$: there is no correlation between the two variables (r=0);
- Alternative hypothesis $\left(H_{1}\right)$: there is a significant correlation between the two variables (r≠0).

A correlation matrix was constructed, with each element representing the correlation between a dynamic variable and a static metric. Based on the values of the correlation coefficients, the following significance notation rules were applied:

***: p<0.001 (highly significant);
**: p<0.01 (moderately significant);
*: p<0.05 (significant);
- No marking: no significant correlation.

## 4. Results

This section investigates the relationships between dynamic gait characteristic variables and static body metrics. Among the dynamic gait characteristic variables, a primary focus is placed on center displacement, and specifically its mean directional shift component, which serves as a proxy for movement variability. By analyzing these correlations, we seek to explore potential physiological factors that may be related to movement behavior. The results presented in this chapter contribute to understanding how body composition and physical characteristics influence movement patterns.

**Figure 8:**
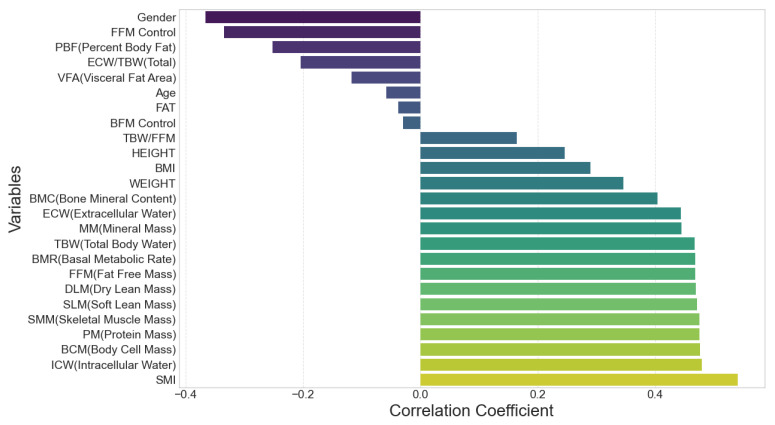
Correlation of biometric variables with mean_directional_shift.

The correlation analysis was conducted using *Pearson correlation coefficients* to evaluate the linear relationships between the mean directional shift and a range of biometric variables, such as body water composition, muscle mass, and body fat percentage. After a Pearson correlation analysis was conducted, the strength of the correlation between variables was determined using the absolute value of the correlation coefficient, while the statistical significance of the correlation was assessed using *p*-values. The thresholds for statistical significance were set as follows: *p* < 0.05 indicating marginal significance, *p* < 0.01 indicating moderate significance, and *p* < 0.001 indicating strong significance. The detailed tabular data summarizing these correlations is included in Table 6. To aid interpretation, a ranking of correlation coefficients is visualized in Figure 8.

As shown in Table 7, prior to conducting the correlation analysis, the study first performed a simple linear regression test on each body metric, with the horizontal directional shifts in center displacement (denoted as “mean_directional_shift”) serving as the dependent variable, and the results are presented herein. It can be seen from the regression results that the significance of different metrics varies (e.g., *ICW* and *PM* show significant correlations, while *FAT* and *BMI* show no significant correlations). However, it should be noted that, due to the limited sample size of this study, the significance results from the linear regression can only be used as a reference and cannot completely rule out the possibility of potential associations. Future studies will further verify the relationships between the aforementioned variables by expanding the sample size.

### 4.1. Result 1: Overview of Correlation

The analysis identified correlations between the mean directional shift and several biometric variables, highlighting the interplay between body composition and movement variability. The correlations ranged from moderately strong positive associations to negligible or nonsignificant relationships, reflecting the heterogeneous influence of physiological factors on movement dynamics. As shown in Table 6, *SMM* (ρ=0.496, p<0.01): this indicates that individuals with greater skeletal muscle mass exhibit more pronounced horizontal directional shifts, suggesting a potential direct positive link between muscular capacity and movement variability. *SLM* (ρ=0.495, p<0.01) and *FFM* (ρ=0.493, p<0.01): these measures of lean body mass were also significantly positively associated with horizontal directional shifts, potentially reinforcing the importance of muscle integrity and strength in dynamic movement. *BCM* (ρ=0.497, p<0.01): a strong positive correlation was observed with body cell mass, which reflects the metabolically active portion of the body, potentially underscoring its role in enabling effective and varied movement. *SMI* (ρ=0.561, p<0.01): the strongest positive correlation was observed with *SMI*, a composite measure of skeletal muscle relative to height, suggesting a potential key role of proportional muscularity in directional movement.

Additionally, it can be observed from the analysis results in Table 6 that the scores of the *SPPB* exhibited no significant correlations with multiple biometric variables. This may, to a certain extent, explain why the *SPPB* test was unable to reflect physical characteristics such as *SMI* in older adults, making it difficult to identify relevant associations. In contrast, mean directional shift may be more effective in accurately reflecting the physiological status of the elderly.

### 4.2. Result 2: Correlation Analysis Between mean_directional_shift and SPPB

**Figure 9:**
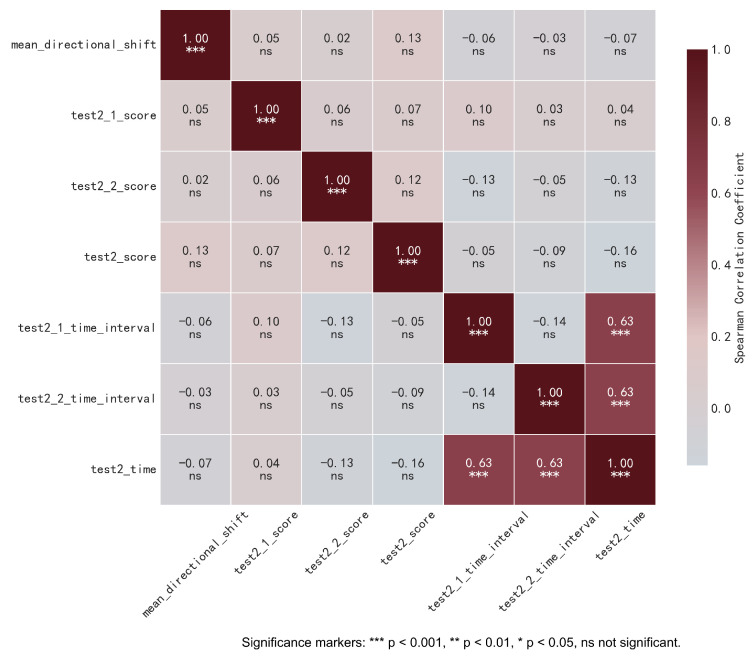
Spearman correlation analysis between mean_directional_shift and SPPB.

As shown in Figure 9, the Spearman correlation analysis revealed no correlation between mean_directional_shift and either the scores or time taken for SPPB Test 2 (walking test). Combined with the findings in Table 6, where SPPB Test 2 scores also exhibited correlations with multiple biometric variables, this allows for the inference that SPPB Test 2 may face limitations in accurately reflecting the physiological status of older adults. This limitation likely arises because Test 2 only focuses on the time required to complete the test, without specifically capturing changes in the movement characteristics of older adults during walking. Meanwhile, the widespread strong correlations observed in Table 6 between mean_directional_shift and multiple biometric variables suggest that mean_directional_shift may serve as a more appropriate method for monitoring the movement and physiological status of older adults. Among the SPPB subtests, Test 2 was selected as the primary focus for analysis for the following reasons: In Test 1 (single-leg standing balance test), the center of pressure displacement was too small to provide meaningful insights; additionally, Test 3 (chair stand balance test) and Test 1 failed to adequately reflect the motor function status of older adults. Thus, the final analysis centered on SPPB Test 2.

### 4.3. Result 3: Negative and Insignificant Correlations

In contrast, certain variables exhibited negative or negligible associations with mean directional shift; specific data are detailed in Table 6: *PBF* (ρ=−0.256, p>0.05): greater body fat percentages were associated with reduced movement variability, aligning with the notion that excess adiposity may constrain physical dynamics. *FAT* (ρ=−0.045, p>0.05): similar to *PBF*, fat mass showed a minimal negative relationship with directional shift, though the association was not statistically significant. Extracellular water-to-*total body water ratio (ECW/TBW)* (ρ=−0.122, p>0.05): the weak correlation suggests that the distribution of water within the body has a limited direct impact on directional movement. The results suggest that measures of lean mass, particularly skeletal muscle mass and indices such as *SMI*, are critical predictors of movement variability. This aligns with biomechanical theories that emphasize the role of muscle strength and control in facilitating complex motor tasks. Conversely, the negligible associations with fat mass and body fat percentage highlight the limited functional contribution of adipose tissue to movement dynamics.

### 4.4. Result 4: Gender Differences in Correlation Analysis

**Figure 10:**
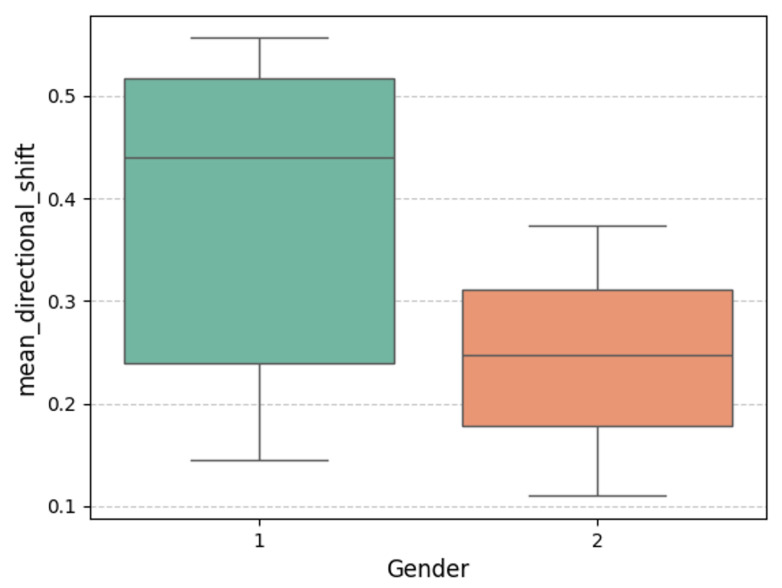
Boxplot of mean_directional_shift by gender.

**Figure 11:**
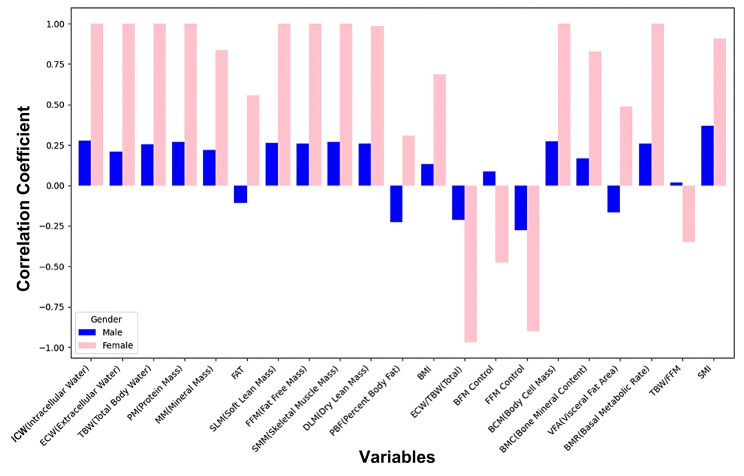
Correlation of variables with mean_directional_shift by gender.

The correlation between *mean_directional_shift* (the average angular change in the center of mass during movement) and various body metrics exhibited significant differences between male and female groups. The analysis below evaluates these differences from the perspectives of specific variables and gender-based variations, concluding with a comprehensive assessment. It should be noted that the total number of participants in this analysis was relatively small (19 in total), with a gender ratio imbalance (16 males to 3 females). Due to these limitations, the analysis results should be regarded as reference information only and interpreted with caution. A boxplot and correlation of variables of mean_directional_shift by gender are shown in Figure 10 and Figure 11, respectively.

#### 4.4.1. Correlation Analysis in Males

As shown in Figure 11, in the male group, variables such as *ICW*, *ECW*, *TBW*, and *SMI* showed positive correlations with *mean_directional_shift*. This indicates that an improved water balance and increased muscle mass may enhance the dynamic adjustment capacity in males. Furthermore, *SLM* and *SMM*, which reflect overall muscle content, are also positively correlated with *mean_directional_shift*, emphasizing the critical role of muscle mass in dynamic movement.

Conversely, fat-related variables such as *PBF* and *FAT* exhibit weaker or even negative correlations, suggesting that excessive body fat may hinder dynamic performance. Functional variables such as *fat-free mass control (FFM Control)* and *body fat mass control (BFM Control)* show weaker correlations, implying that direct effects of muscle and fat mass on center of mass changes are more critical during dynamic movement.

#### 4.4.2. Correlation Analysis in Females

As shown in Figure 11, in the female group, muscle-related metrics such as *SMI*, *SMM*, and *SLM* displayed stronger positive correlations with *mean_directional_shift*. Meanwhile, as shown in Figure 10, the average directional shift distance of females was smaller than that of males, and both the minimum and maximum directional shift distances of females were also smaller than those of males. This phenomenon may indicate that the basic motor ability of elderly females was less than that of males. This highlights the importance of increasing muscle mass in improving females’ dynamic movement capacity, potentially due to the generally lower baseline muscle mass in females.

Additionally, *BMI* and *PBF* show more pronounced positive correlations, suggesting that moderate increases in body weight and body fat may positively influence dynamic adjustment capacity, likely because these metrics were associated with strength improvements. The ratio of extracellular water to *ECW/TBW* exhibited a negative correlation, which was more pronounced in females, potentially indicating the impact of fluid distribution imbalance on movement ability. Similar to that in males, *FFM* control and *BFM* control showed weaker correlations, suggesting that overall muscle and fat mass may exert more direct effects on dynamic movement.

### 4.5. Result 5: Comprehensive Analysis

The correlation patterns between *mean_directional_shift* and body metrics reveal significant gender differences (see Figure 11). In males, dynamic movement is more directly influenced by water balance and muscle mass, while in females, both muscle mass and body fat may play dual roles in dynamic capacity. Additionally, the positive correlation of certain metrics in females may reflect a compensatory effect of body weight on dynamic ability when muscle mass is lower.

The analysis underscores the critical importance of muscle-related metrics in both genders, with females exhibiting a greater dependency on these metrics. On the other hand, fat-related metrics demonstrated a positive role in females but may impede dynamic ability in males. This suggests that strategies to improve physical capacity in older adults should consider gender differences, focusing on increasing muscle mass and optimizing fat distribution to achieve better dynamic movement capabilities.

Moreover, the analysis of water-balance-related variables further highlights the significance of hydration in the health of older adults, providing valuable insights for designing targeted interventions in the future.

### 4.6. Result 6: Visualization and Interpretation

Figure 8 provides a ranked visualization of the correlation coefficients, facilitating a comparative interpretation of variable influences. The positive contributions of lean mass metrics are clearly evident, with *SMI*, *SMM*, and *BCM* occupying the highest ranks. Variables with negligible or negative correlations, such as *PBF* and *ECW/TBW*, are positioned toward the lower end of the spectrum. This graphical representation underscores the dominant role of muscle-related metrics in explaining directional shift variability while de-emphasizing the influence of adiposity and water distribution. The consistent pattern observed across multiple measures of lean mass strengthens the reliability of these findings.

### 4.7. Discussion

The global acceleration in population aging has led to a substantial increase in the demand for eldercare services and health-monitoring systems, placing considerable pressure on both public medical infrastructure and family caregiving systems. Against this backdrop, ensuring efficient assessment and the continuous monitoring of elderly individuals’ health status emerged as a critical societal challenge. However, traditional mobility assessment methods involve obvious limitations. On the one hand, they relied on contact-based measurement techniques, which were invasive and impractical for continuous monitoring in daily scenarios. On the other hand, most existing studies addressed gait performance and physiological indicators as distinct research domains, resulting in a limited understanding of their interrelated mechanisms. Although several studies had attempted to bridge this gap by incorporating musculoskeletal modeling with joint kinematics, such efforts were typically confined to disease-specific diagnostic contexts or narrowly defined participant populations. Moreover, mainstream tools like the *SPPB* only focused on a single dimension and failed to capture dynamic features such as changes in the body’s center of mass during walking. To address these limitations, the present study proposed and validated an integrated sensor instrumentation framework that integrated non-invasive dynamic gait analysis and static body composition assessment. This framework extracted dynamic gait features, including mean directional shift and center displacement, using the computer vision model *PoseNet*, and combined them with body composition indicators measured via the *InBody* bioelectrical impedance device, such as skeletal muscle index and skeletal muscle mass, to analyze their correlations. It not only verified the potential of dynamic metrics in assessing muscle function and the early detection of sarcopenia but also reduced the dependence on laboratory-grade instrumentation. This provided an affordable and scalable technical solution for the continuous health monitoring of older adults in community and home settings, contributing to the development of intelligent eldercare environments.

#### 4.7.1. Interpretation of Results

We highlight the importance of muscle mass and strength in motor performance. The strong associations with *SMI* and *SMM* are particularly noteworthy, suggesting that proportional and absolute measures of muscle might be equally critical. These findings could have practical implications for rehabilitation and athletic training, where interventions aimed at improving lean mass could potentially enhance movement variability and adaptability. The lack of significant correlations with fat-related variables further supports the view that adipose tissue is likely to play a secondary role in dynamic movement. This distinction underscores the need for targeted approaches in movement-related studies, focusing on muscle-specific metrics, rather than generalized body composition measures.

This study analyzed the relationship between *mean_directional_shift* (the average angular change in the center of mass during movement) and various body metrics, including the *SMI*, to reveal the characteristics of elderly individuals during movement and their potential connection to health status. The *mean_directional_shift* may reflect the flexibility and motor control of elderly individuals during walking or other dynamic movements. The results showed a significant positive correlation between *mean_directional_shift* and SMI, indicating that elderly individuals with greater angular changes in direction tend to have a greater skeletal muscle index value. According to the definition of *SMI*, the health standard is *SMI* ≥ 7.0 kg/m^2^ for men and ≥ 5.7 kg/m^2^ for women. An increase in *mean_directional_shift* is likely to reflect better muscle strength and motor performance. Individuals with greater *mean_directional_shift* could experience advantages in dynamic balance, flexibility, and coordination, consistent with the limb muscle strength indicated by *SMI*. The analysis of variables positively correlated with *mean_directional_shift*, apart from *SMI*, *SMM*, and *ICW*, also showed significant positive correlations. This result further supports the hypothesis that *mean_directional_shift* may reflect comprehensive motor functions, such as muscle strength and physical flexibility. These findings align with previous studies and underscore the pivotal role of skeletal muscles in maintaining motor abilities in older adults [65].

On the other hand, *mean_directional_shift* was significantly negatively correlated with fat-related variables, such as *PBF* and *VFA*. This suggests that elderly individuals who exhibit frequent directional changes during movement tend to have a lower fat mass. This phenomenon may arise because movements involving large directional changes (e.g., turning, step adjustments) require greater muscle engagement, while greater fat mass could potentially impede such flexible movements. As a key indicator of muscle mass and function in older adults, *SMI* can effectively reflect overall health status. An increase in *SMI* is typically accompanied by stronger limb muscle mass and improved balance, and its correlation with *mean_directional_shift* highlights their synergistic role in potentially reflecting motor health. Therefore, *mean_directional_shift* could serve as a potential dynamic indicator for monitoring changes in *SMI* and assessing the health status of elderly individuals [66]. In terms of health interventions, elderly individuals with lower *mean_directional_shift* values can improve their motor flexibility and health status by enhancing muscle strength, such as increasing *SMI* and *SMM*. By combining the monitoring of *mean_directional_shift* and *SMI*, a more comprehensive and dynamic assessment tool for health management systems could potentially be developed, enabling personalized health interventions.

In summary, the significant correlation between *mean_directional_shift* and health metrics such as *SMI* underscores its importance in evaluating the health status of elderly individuals. Future research could incorporate longitudinal data to further explore the dynamic relationship between *mean_directional_shift* and *SMI*, as well as their potential links to health outcomes in older adults, such as fall risk and mobility decline. This would provide a more comprehensive scientific basis for health monitoring and intervention in older adults [67]. This section highlights the critical role of lean mass, particularly skeletal muscle, in influencing directional movement metrics. Table 6 presents detailed results, providing a comprehensive understanding of the interaction between biometric variables and movement variability.

#### 4.7.2. Limitations of the Study

Several limitations of this study merit attention. First, the study was constrained by a relatively small sample size (only 19 participants) and an unbalanced data structure; for instance, 80% of the participants were male, and 50% fell into the 80–89-year-old age group. It is important to note that, although this study adopted a fixed experimental environment design—which played a positive role in enhancing data comparability within the study cohort—it simultaneously reduced experimental flexibility. The fixed environmental conditions may affect the reproducibility of the study results across different scenarios, making it difficult to fully verify the applicability of the findings in diverse real-world contexts. This gender imbalance, with a disproportionately high male representation, poses two key challenges: on the one hand, stratified analyses by gender lack sufficient statistical power and reference value due to the small sample size of female participants; on the other hand, the sample recruitment was primarily based on the accessibility of elderly individuals in local community centers (where male participants were more willing to participate in physical measurement assessments), which may have further narrowed the sample’s representativeness. This overall unbalanced structure may restrict the generalizability of the findings and elevate the risk of sampling bias, as the limited and skewed sample fails to fully represent the broader elderly population with diverse demographic characteristics, thereby impeding a comprehensive delineation of the correlation patterns between gait dynamics and body composition across elderly individuals of different genders and age brackets. Additionally, potential inadequacies exist in the data processing workflow, primarily in the alignment of motion feature data. The matching precision of dynamic gait data and static body composition data in terms of temporal and dimensional aspects requires enhancement, and such imprecision may introduce errors that could compromise the reliability of subsequent correlation analysis results. Another notable limitation relates to potential multicollinearity among body composition features. For example, variables like body fluid content and fat mass may exhibit inherent correlations, which could inflate the variance of regression coefficients in linear models and lead to unstable parameter estimates. While this study did not explicitly quantify or address multicollinearity (e.g., via variance inflation factor [VIF] detection or dimensionality reduction techniques such as principal component analysis [PCA]), future studies could incorporate linear regression models with multicollinearity correction (e.g., ridge regression) to disentangle the independent contributions of correlated features and clarify the specific relationships between individual body composition indicators and gait dynamics. Furthermore, the exclusive reliance on a Pearson correlation analysis to explore inter-variable relationships entails inherent limitations. This method solely captures linear correlations and may miss non-linear associations between dynamic gait metrics (e.g., mean directional shift) and static body composition indicators. In addition, regarding the issue of multiple comparisons, this study did not adopt formal correction methods (e.g., Bonferroni correction or Benjamini–Hochberg procedure). This choice is justified by the exploratory nature of the research: the primary goal was to identify preliminary correlation patterns between gait dynamics and body composition in the elderly, rather than to confirm definitive, statistically corrected associations. As such, the uncorrected results should be interpreted as tentative hypotheses for future confirmatory studies with larger sample sizes. As a result, the combined limitations of Pearson analysis and uncorrected multiple comparisons might overlook crucial complex relationships within the data and fail to fully unravel the intrinsic connections between the two sets of variables.

## 5. Conclusions

Traditional gait analysis methods often depend on laboratory-based systems, such as motion-capture cameras, force plates, and wearable sensors. Although these tools offer excellent measurement precision, their prohibitive cost, complexity of operation, and limited adaptability to daily environments restrict their widespread application in geriatric health monitoring. In response to these limitations, this study innovatively adopted a multi-sensor fusion strategy, introducing a contactless assessment framework that integrated dynamic gait features extracted from video data with static body composition indicators. This approach combined *PoseNet*’s non-contact visual sensing, which captures dynamic gait, with *InBody*’s bioelectrical impedance, used to measure static body composition. By doing so, it enables a more holistic health assessment than single modality approaches and seeks to support the development of intelligent, continuous, and personalized monitoring systems for older adults.

By applying the *PoseNet* model to gait videos recorded during the *SPPB*, we extracted two key indicators, center displacement and mean directional shift—which reflect postural control and stability during walking. These visual features were statistically examined in relation to body composition variables obtained from *InBody* measurements, including *SMM*, *SMI*, and *FFM*.

The results show that the mean directional shift is positively associated with muscle-related indicators such as *SMI* (ρ=0.561, p<0.01) and *SMM* (ρ=0.496, p<0.01), while it is negatively associated with fat-related measures such as *PBF* and *VFA*. These findings suggest that directional variability in gait may serve as an informative proxy for muscle strength and neuromuscular coordination. In contrast to conventional gait assessment methods that focus on lower-limb function, our approach demonstrates the feasibility of using upper-body movement patterns to infer internal muscular conditions. Overall, the proposed multi-sensor fusion method demonstrates how visual gait features (extracted via the low-cost, easily deployable PoseNet), combined with body composition data, may be able to create more accessible tools for evaluating physical function in older adults. This integration enhances the ability to detect early signs of sarcopenia and other age-related declines by linking dynamic movement patterns to underlying physiological states.

## Figures and Tables

**Table 1:** Camera and experimental setup parameters.

Category	Description
Camera Settings	Sanwa Supply webcam; 1MP CMOS sensor; resolution up to 1280 × 720 (720 p); video formats YUY2/MJPEG; ultra wide-angle 150° lens (F1.75, f = 2.5 mm); frame rate up to 30 fps; manual focus; automatic adjustment of brightness, contrast, and white balance; minimum illumination ≥30 Lux; USB 2.0 interface, UVC compliant; built-in microphone (PCM audio)
Environment Setup	Controlled laboratory environment with stable lighting; plain background; participants wore light, non-loose clothing and avoided accessories
Recording Angles	Mainly frontal view combined with slight sagittal view to capture stride length and center of mass changes
Spatio-Temporal Resolution	Spatial resolution: 1280 × 720 pixels (approx. 2–3 mm/pixel depending on distance); Temporal resolution: up to 30 fps
Evaluation Metrics	Pose confidence: mean 0.59–0.68, max 0.83;
	Frame drop rate: ∼5% abnormal frames removed;
	Filtering: 95% percentile threshold (cut-off = 9.42 pixels);
	Data interpolation: linear interpolation for missing frames;
	Tracking success rate: continuous keypoint tracking >90%

**Table 2:** Demographic and lifestyle characteristics of the participants in the experiment.

Demographic Characteristics		Frequency	%
Gender	Male	16	80
	Female	4	20
Total		20	100
AgeGroup	80∼89	10	50
	60∼69	6	30
	70∼79	4	20
Total		20	100
LivingAlone	Not	19	95
	Yes	1	5
Total		20	100
Spouse	Married	18	90
	Single	2	10
Total		20	100
Employment	Working	14	70
	Working	6	30
Total		20	100

Note: One participant withdrew from the data collection process midway due to personal reasons.

**Table 3:** Definitions of InBody body composition indicators.

Indicator Abbreviation	Full English Name	Definition
ICW (L)	Intracellular Water	Water inside cell membranes (80% of ICF); reduced in aging or malnutrition (unit: Liter, L).
ECW (L)	Extracellular Water	Water outside cell membranes (98% of ECF); abnormally high in edematous diseases.
TBW (L)	Total Body Water	Sum of ICW and ECW; transports nutrients/waste, indicating metabolic status.
PM (kg)	Protein Mass	Main muscle component (with water); insufficiency means poor cellular nutrition (unit: Kilogram, kg).
MM (kg)	Mineral Mass	80% in bones (body support); deficiency raises osteoporosis/fracture risks, correlates with FFM.
FAT (kg)	Body Fat	Excess energy stored in fat cells; excess causes obesity and related issues.
SLM (kg)	Skeletal Muscle Mass of Limbs	Limb-specific SMM subset; critical for gait and movement.
FFM (kg)	Fat-Free Mass	Weight without fat (muscles, bones, organs, fluids); equals muscle mass + BMC.
SMM (kg)	Skeletal Muscle Mass	Voluntary striated muscle; excludes visceral/cardiac muscle, core for movement function.
DLM (kg)	Dry Lean Mass	FFM without water; reflects solid non-fat components (e.g., protein, minerals).
PBF (%)	Percent Body Fat	BFM/total weight ratio; normal: 10–20% (male), 18–28% (female) (unit: Percent, %).
BMI (kg/m^2^)	Body Mass Index	Weight (kg)/height^2^ (m^2^); normal range: 18.5–25.0 kg/m^2^ (WHO) (unit: Kilogram per square meter, kg/m^2^).
ECW/TBW (Ratio)	Extracellular Water/ Total Body Water	ECW/TBW ratio (0.380 in healthy people); >0.400 = possible edema, used for hemodialysis DW estimation (unit: Ratio, no dimensional unit).
BFM (kg)	Body Fat Mass	Total fat tissue mass; stored unutilized energy, key for obesity assessment.
BCM (kg)	Body Cell Mass	Fat-free cell mass (muscle, viscera, blood); protein mass + ICW, reflecting nutrition/function.
BMC (kg)	Bone Mineral Content	Total bone minerals (80% of body minerals); derived vie FFM–muscle mass.
VFA (cm^2^)	Visceral Fat Area	Fat around internal organs; excess raises metabolic disease risks (e.g., diabetes) (unit: Square centimeter, cm^2^).
BMR (kcal)	Basal Metabolic Rate	Minimum energy for resting life activities; InBody S10 calculates via FFM (unit: Kilocalorie, kcal).
TBW/FFM (%)	Total Body Water/ Fat-Free Mass	TBW/FFM ratio (73% in healthy people); reflects FFM water saturation, deviates in malnutrition/disease.
SMI (kg/m^2^)	Skeletal Muscle Index	SMM/height^2^ (m^2^); sarcopenia diagnostic: <7.0 kg/m^2^ (male), <5.7 kg/m^2^ (female) (AWGS 2019) (unit: Kilogram per square meter, kg/m^2^).

**Table 4:** Descriotive statistics of InBody indicators (N = 19).

Indicator Abbreviation	mean	std	min	max
ICW (Intracellular Water)	21.216	3.863	13.7	26.3
ECW (Extracellular Water)	13.8	2.446	9	17.1
TBW (Total Body Water)	35.016	6.288	22.7	43.4
PM (Protein Mass)	9.163	1.67	5.9	11.4
MM (Mineral Mass)	3.246	0.575	2.07	4.2
FAT	11.811	5.794	3.7	26.2
SLM (Soft Lean Mass)	44.721	8.066	28.9	55.5
FFM (Fat Free Mass)	47.416	8.507	30.7	58.8
SMM (Skeletal Muscle Mass)	25.653	5.025	15.8	32.3
DLMM (Dry Lean Mass)	12.4	2.227	8	15.5
PBF (Percent Body Fat)	19.7	8.228	7.8	32.8
BMI	22.695	2.933	17.4	30.5
ECW/TBW (Total)	0.394	0.007	0.358	0.41
BFM Control	−2.421	5.507	−18.5	5.2
FFM Control	1.679	2.525	0	6.8
BCM (Body Cell Mass)	30.384	5.534	19.6	37.7
BMC (Bone Mineral Content)	2.687	0.465	1.76	3.51
VFA (Visceral Fat Area)	46.316	21.765	17	92.7
BMR (Basal Metabolic Rate)	1394.368	183.521	1034	1640
TBW/FFM	73.789	0.38	72.9	74.4
SMI	8.132	1.322	5.5	10.2

Note: This table summarizes key body composition metrics measured by InBody for 19 participants.

**Table 5:** Normality test results of body composition variables.

Variable	Mean ± SD	Skewness	Kurtosis	Shapiro–Wilk Statistic	*p*-Value	Normality
ICW	21.24 ± 3.67	−0.5916	−0.3995	0.9415	0.3360	Conforms
ECW	13.86 ± 2.33	−0.6855	−0.2418	0.9464	0.4024	Conforms
TBW	35.09 ± 5.98	−0.6453	−0.3280	0.9441	0.3699	Conforms
PM	9.17 ± 1.59	−0.5812	−0.4208	0.9462	0.3994	Conforms
MM	3.24 ± 0.52	−0.4442	−0.0930	0.9624	0.6769	Conforms
FAT	11.76 ± 6.12	0.9304	0.4521	0.9270	0.1936	Conforms
SLM	44.81 ± 7.67	−0.6385	−0.3326	0.9432	0.3578	Conforms
FFM	47.49 ± 8.06	−0.6246	−0.3466	0.9442	0.3717	Conforms
SMM	25.68 ± 4.78	−0.5973	−0.3908	0.9417	0.3387	Conforms
DLM	12.40 ± 2.09	−0.5556	−0.4003	0.9503	0.4613	Conforms
PBF	19.46 ± 8.42	0.4002	−1.1827	0.9170	0.1312	Conforms
BMI	22.54 ± 2.99	0.9122	2.1890	0.9495	0.4494	Conforms
ECW/TBW Ratio	0.40 ± 0.01	0.7134	0.4253	0.9507	0.4672	Conforms
BFM Control	−2.50 ± 5.82	−0.8938	0.3404	0.9299	0.2165	Conforms
FFM Control	1.85 ± 2.62	1.1534	−0.2033	0.7153	0.0002	Does not conform
BCM	30.41 ± 5.26	−0.5927	−0.4068	0.9416	0.3371	Conforms
BMC	2.68 ± 0.42	−0.2622	−0.1333	0.9598	0.6269	Conforms
VFA	46.99 ± 22.83	0.8531	−0.1481	0.9124	0.1099	Conforms
BMR	1396.00 ± 173.80	−0.6220	−0.3484	0.9443	0.3726	Conforms
TBW/FFM Ratio	73.83 ± 0.38	−0.7117	1.0523	0.9414	0.3353	Conforms
SMI	8.10 ± 1.25	−0.8427	0.0216	0.9167	0.1300	Conforms
Age	77.88 ± 7.76	−0.6733	−0.7516	0.9048	0.0818	Conforms
Height	161.82 ± 8.19	−0.6129	−0.3191	0.9360	0.2735	Conforms
Weight	59.25 ± 10.24	0.3329	1.8835	0.9254	0.1819	Conforms
mean_directional_ shift	0.36 ± 0.15	−0.5787	−0.726	0.9339	0.2527	Conforms

**Table 6:** Correlation between biometric variables and movement metrics.

Variable	mean_ directional_shift	mean_ displacement	test2_1_score	test2_2_score	test2_score
ICW (Intracellular Water)	0.409 ** (0.042)	−0.014 (0.959)	−0.091 (0.728)	0.166 (0.524)	−0.115 (0.661)
ECW (Extracellular Water)	0.480 * (0.051)	0.037 (0.887)	−0.107 (0.684)	0.131 (0.616)	−0.143 (0.583)
TBW (Total Body Water)	0.493 ** (0.044)	0.006 (0.981)	−0.098 (0.710)	0.153 (0.558)	−0.126 (0.629)
PM (Protein Mass)	0.494 ** (0.044)	−0.001 (0.996)	−0.097 (0.711)	0.172 (0.510)	−0.122 (0.640)
MM (Mineral Mass)	0.485 ** (0.048)	−0.005 (0.983)	−0.177 (0.498)	0.274 (0.288)	−0.153 (0.588)
FAT	−0.045 (0.863)	0.041 (0.875)	−0.262 (0.399)	−0.081 (0.759)	−0.278 (0.279)
SLM (Soft Lean Mass)	0.495 ** (0.043)	0.003 (0.991)	−0.094 (0.721)	0.155 (0.553)	−0.122 (0.642)
FFM (Fat Free Mass)	0.493 ** (0.044)	0.004 (0.988)	−0.102 (0.696)	0.165 (0.528)	−0.127 (0.627)
SMM (Skeletal Muscle Mass)	0.496 ** (0.043)	−0.014 (0.957)	−0.093 (0.723)	0.166 (0.523)	−0.115 (0.659)
DLM (Dry Lean Mass)	0.492 ** (0.045)	−0.002 (0.993)	−0.116 (0.657)	0.197 (0.448)	−0.129 (0.623)
PBF (Percent Body Fat)	−0.256 (0.321)	0.066 (0.802)	−0.252 (0.323)	−0.116 (0.659)	−0.252 (0.329)
BMI	0.261 (0.312)	0.097 (0.711)	−0.193 (0.458)	0.221 (0.393)	−0.192 (0.460)
ECW/TBW Ratio	−0.122 (0.642)	0.330 (0.195)	−0.036 (0.890)	−0.280 (0.276)	−0.114 (0.663)
BFM Control	−0.005 (0.983)	−0.098 (0.707)	0.142 (0.585)	0.126 (0.631)	0.169 (0.516)
FFM Control	−0.296 (0.249)	−0.073 (0.782)	0.170 (0.515)	−0.744 *** (0.001)	0.117 (0.653)
BCM (Body Cell Mass)	0.497 ** (0.043)	−0.013 (0.960)	−0.094 (0.721)	0.168 (0.515)	−0.117 (0.655)
BMC (Bone Mineral Content)	0.447 * (0.072)	0.011 (0.965)	−0.243 (0.346)	0.315 (0.218)	−0.209 (0.420)
VFA (Visceral Fat Area)	−0.132 (0.615)	0.069 (0.793)	−0.244 (0.346)	−0.140 (0.593)	−0.275 (0.286)
BMR (Basal Metabolic Rate)	0.493 ** (0.044)	0.004 (0.989)	−0.104 (0.692)	0.165 (0.526)	−0.128 (0.624)
TBW/FFM Ratio	0.174 (0.504)	0.188 (0.471)	0.258 (0.318)	−0.405 (0.107)	0.082 (0.755)
SMI	0.561 ** (0.019)	0.130 (0.619)	0.011 (0.965)	0.182 (0.485)	−0.004 (0.987)

Significance markers: *** *p* < 0.001, ** *p* < 0.01, * *p* < 0.05, ns not significant.

**Table 7:** Simple linear regression results (dependent variable: mean_directional_shift).

Variable	Constant Coefficient	Variable Coefficient	t-Statistic	Significance
ICW	−0.0817	0.0209	2.2282	*
ECW	−0.0775	0.0318	2.1185	ns
TBW	−0.0834	0.0127	2.1956	*
PM	−0.0759	0.0478	2.2021	*
MM	−0.1011	0.1432	2.1501	*
FAT	0.3762	−0.0011	−0.1761	ns
SLM	−0.0827	0.0099	2.2063	*
FFM	−0.0851	0.0094	2.1970	*
SMM	−0.0472	0.0160	2.2110	*
DLM	−0.0863	0.0362	2.1891	*
PBF	0.4540	−0.0047	−1.0267	ns
BMI	0.0599	0.0134	1.0473	ns
ECW/TBW	1.4680	−2.7969	−0.4748	ns
BFM Control	0.3624	−0.0001	−0.0212	ns
FFM Control	0.3948	−0.0174	−1.2000	ns
BCM	−0.0793	0.0145	2.2157	*
BMC	−0.0794	0.1651	1.9367	ns
VFA	0.4044	−0.0009	−0.5139	ns
BMR	−0.2476	0.0004	2.1964	*
TBW/FFM	−4.8659	0.0708	0.6850	ns
SMI	−0.1993	0.0694	2.6252	*
Age	0.4518	−0.0011	−0.2237	ns
HEIGHT	−0.6314	0.0061	1.3395	ns
WEIGHT	0.0409	0.0054	1.5001	ns

Significance markers: * *p* < 0.05, ns not significant.

## Data Availability

The original contributions presented in this study are included in the article. Further inquiries can be directed to the corresponding author(s).
